# What we can learn from deep space communication for reproducible bioimaging and data analysis

**DOI:** 10.1038/s44320-023-00002-9

**Published:** 2023-12-13

**Authors:** Tatiana Woller, Christopher J Cawthorne, Romain Raymond Agnes Slootmaekers, Ingrid Barcena Roig, Alexander Botzki, Sebastian Munck

**Affiliations:** 1 VIB Technology Training, Data Core, and VIB BioImaging Core, Ghent & Leuven, Ghent, Belgium; 2 Department of Neuroscience, KU Leuven, Leuven, Belgium; 3 Department of Imaging and Pathology, Nuclear Medicine and Molecular Imaging, KU Leuven, Leuven, Belgium; 4 NSANGA, 36 Pakenstraat, Leuven, 3001 Belgium; 5 Support for Research Data Management (RDM), KU Leuven, Leuven, Belgium; 6 VIB Technology Training, Ghent, Belgium; 7 VIB BioImaging Core, Leuven, Belgium

**Keywords:** Methods & Resources, Science Policy & Publishing

## Abstract

This Commentary takes inspiration from space communication which uses error-correction protocols based on redundant data sources and discusses how we can use AI-based language models to improve documentation and reproducibility in bioimaging.

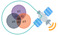

## Teaser

Multiple initiatives have attempted to define and recommend the annotation of images with metadata. However, proper documentation of complex and evolving projects is a difficult task, and the variety of storage methods—electronic labnotebooks, metadata servers, repositories and manuscripts—along with data from different time points of a given project leads to either redundancy in annotation or omissions. In this Commentary, we discuss how to tackle this problem, taking inspiration from space communication which uses error-correction protocols based on redundancy for data transmission. We provide a proof of concept using an Artificial Intelligence (AI) language model to digest redundant metadata entries of this manuscript and visualize the differences to complete metadata entries, highlight inconsistencies and correct human error to improve the documentation for more reproducibility and reusability.

## The reproducibility issue and related initiatives

The reproducibility crisis—many scientific studies are difficult or impossible to reproduce—threatens science’s very fabric and public credibility. A survey conducted by *Nature* in 2016 showed that more than 70% of researchers did not succeed in reproducing someone else’s experiments; more than half could not reproduce their own experiment (Baker, 2016). This failure to reproduce experiments is often attributed to multiple factors, most commonly a lack of access to raw data, insufficient documentation and the inability to manage complex datasets (https://www.nature.com/articles/d42473-019-00004-y).

Reproducibility can be easily confounded with replicability, and its definition depends on the research domain. In biomedical research and computational biology, including bioimaging, “reproducibility” indicates the ability to obtain the same results by using the same data and methods, while “replicability” stands for researchers arriving at the same conclusion using their own data and methods (interestingly, the meanings of reproducibility and repeatability are swapped in computer science and microbiology (Plesser, 2018).

Gunderson et al, define four types of reproducibility based on the quality of the documentation (Gundersen and Kjensmo, 2018). The lowest degree of reproducibility is “R1 description” that encompasses a textual description of the experiment. “R2 code” contains the code/workflow and its associated metadata but lacks the original data. “R3 data” refers to the available dataset and the associated metadata without the workflow for creating the metadata. “R4 experiments” is the highest degree of reproducibility with dataset, code and associated documentation.

Addressing reproducibility via documentation inspired multiple initiatives within the field and beyond. These initiatives attempt to standardize the documentation that accompanies the generation of a bioimaging dataset—and by extension other data analysis disciplines. Organizations such as EOSC assert that the quality of data and associated metadata will improve/if it is Findable, Accessible Interoperable, and Reusable (FAIR; Wilkinson et al, 2016), and will enhance the reproducibility of research (https://zenodo.org/records/7515816). The FAIRification of data requires work at many levels from ontology to reproducible analysis pipelines. Recent ontologies such as REMBI (Sarkans et al, 2021), MITI (Schapiro et al, 2022), and EDAM Bioimaging (https://bioportal.bioontology.org/ontologies/EDAM-BIOIMAGING) provide a starting point to report metadata associated with analysis.

The typical and most common tool for documenting experiments is the electronic labnotebook (Myers et al, 2001), which does not necessarily accommodate the aforementioned standardization of metadata like REMBI, MITI or EDAM. These metadata standards are increasingly incorporated with rich file formats (like OME-TIFF and OME -Zarr) or data tools such as OMERO to find and browse images alongside metadata; https://www.openmicroscopy.org/omero/) and ManGO for associating any file with predetermined metadata on modern data management systems like iRODS (https://irods.org/; https://github.com/kuleuven/mango-metadata-schemas).

One of the consequences of using additional data annotations like REMBI, is that they can lead to a fragmentation of metadata. Information about sample preparation and experimental conditions is typically stored in the labnotebook, whilst information about image acquisition and analysis is typically found in the associated metadata file, so there is a variable degree of redundancy when these sources are combined into a framework such as REMBI. However, as anyone who has had to “recover” data about an image from a labnotebook knows, redundancy can be a feature, not a bug. Seemingly cryptic information (time information, cell naming, filter idiosyncrasies, etc.) may allow confirmation of the identity of an image file via probabilistic inference. This suggests that it can be valuable to keep electronic labnotebook entries, imaging metadata, and the final write-up as separate data entries, as they can all be used to reconstruct complete information in the presence of human error or “noise”.

## Using redundant sources to reconstruct complete information: lessons from space communication

The use of redundant sources to deal with noise is a well-established strategy in space communication, which is gaining increasing interest with current international efforts for unmanned space exploration. A central challenge for controlling robotic probes in outer space or on an alien planet is accurate and reliable communication. The finding that information can be accurately transmitted over noisy channels is based on Claude Shannon’s landmark publication (Shannon, 1948). That it can be corrected is based on Richard Hamming’s pioneering invention of the first error-correcting code in 1950 (Hamming, 1950). Today’s telecommunication routinely employs different strategies for redundancy, error detection and correction, including applications for unreliable storage mediums.

The challenge of creating FAIR bioimage data is similar to the problems in deep-space communication. It can be viewed as a transmission to scientists in the future who would find the data useful or to another entity with which direct communication is not possible, such as another lab, or a successor. As with space communication, the chance that information is lost or parts were not passed on properly and hence are unrecoverable, needs to be accounted for.

In space communication, the error correction is intended to address noise due to weak signal strength and distance; for bioimage data, we can consider the “noise” as imperfect user documentation. In addition to information included in publications—theoretically “stand-alone descriptions” containing all the information needed for replication—the lack of a universal/integrated system that leverages electronic labnotebooks and standardized metadata leads to both omission and (unused) redundancy.

Here, we propose to use the redundancy inherent in different sources of data documentation—namely the electronic labnotebooks, metadata fileservers, manuscripts, GitHub resources, images, and so on that comprise the typical “data package” for a bioimaging experiment—to create the most possible complete annotation and enable cross correction if necessary, using a similar conceptual model. This should consolidate the different metadata sources, help to complete missing information, and future-proof data and support documentation for better reproducibility.

## Application of AI

Whilst the call to proofread and consolidate various metadata entries might be noble, it is unlikely that many researchers will adopt it due to time constraints and its tedious nature. Artificial intelligence (AI) based language models are powerful tools for creating structured outputs that can readily take over tedious proofreading tasks for complementing the human part of the documentation. Commonly-available language models such as GPT-4 have already been used for the post-hoc transformation of free-text radiology reports into structured reporting (Adams et al, 2023). Language models can consequently be used to query if a specific diagnosis is present and to “digest” various sources into a structured report for example in the form of a JSON file.

**Figure 1:**
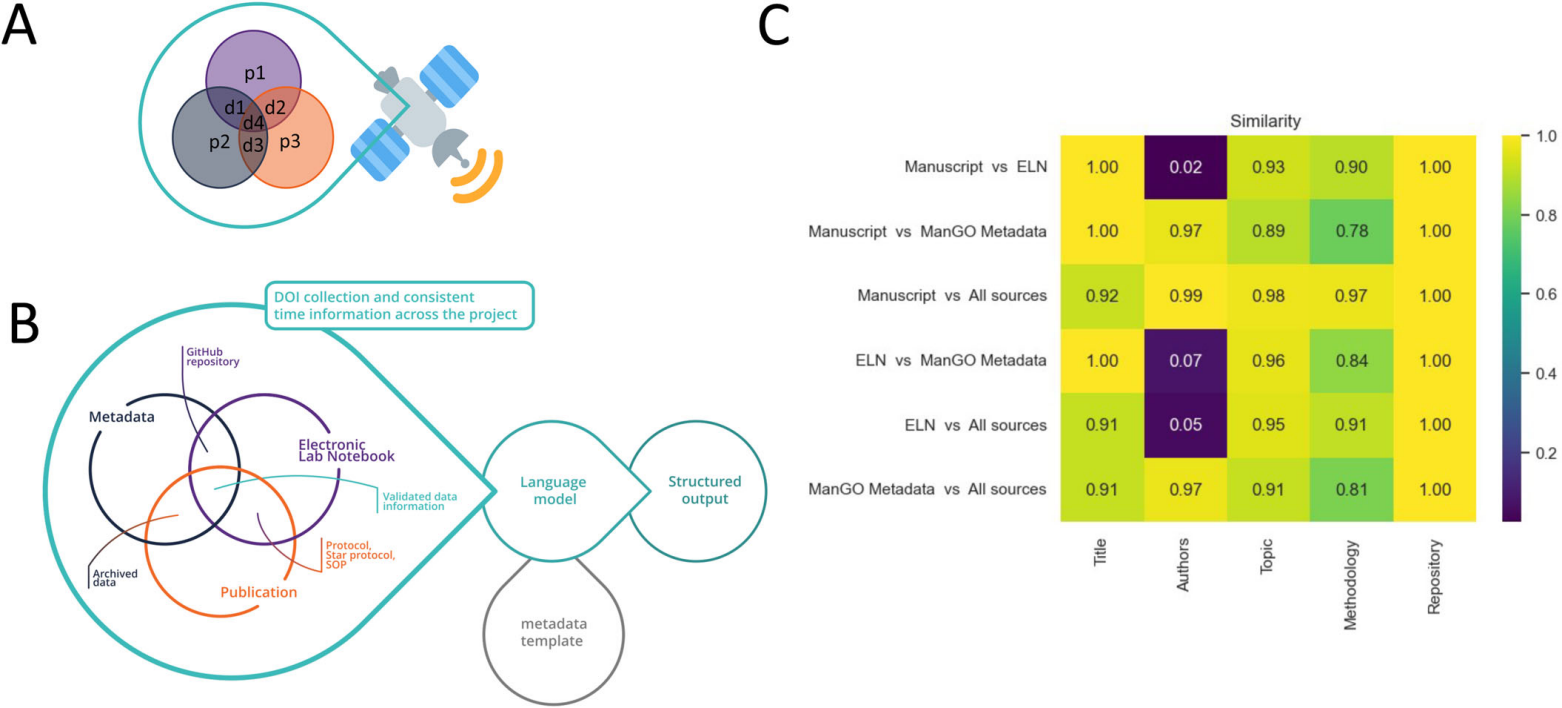
Linking metadata sources and digesting them with language models to generate structured outputs and representations of similarity. (**A**) *Illustration of Hamming code for error correction in data transmission*. Transmission of data (d) and parity (p) bits enables error correction via redundancy (https://en.wikipedia.org/wiki/Hamming_code). (**B**) *Diagram showing the different sources of metadata information and how to bundle them*. Three independent resources—the electronic labnotebook, the data-associated metadata, and the publication—are shown as redundant entries. An AI language model can be used to extract required and standardized data elements for verification, using codewords as a means of error correction analogous to error correction in communication. (**C**) *Heatmap display of similarities between sources by keyword*. A Jupyter notebook using GPT-4 has been used to create a structured output in the form of a CSV file, (see Table 1). The digestion of a labnotebook entry, a metadata file server file, and this manuscript are used to check for keywords. The consistency of the keywords across the sources is displayed in a heatmap using the cosine distance for semantic similarity estimation (https://spacy.io/api/doc).

Such a report can be based on the latest recommendations for metadata such as, for example, REMBI or checklists as proposed by Schmied et al, (2023). Using an AI language model proofreading together with a metadata catalog, can highlight gaps and contradictions and integrate an error correction for the various metadata entries to improve the overall annotation of the data. Redundancy of different (meta) data sources and representation of their consistency can be considered similar to error correction in space communication (Fig. 1A,B). In addition, our analysis offers a feedback on the entries and their completeness.

Such a report could be published alongside a manuscript and could even be a prerequisite for submission. It could be seen as analogous to a preregistered report, where the study proposal is peer-reviewed, only that here the documentation is retrospectively reviewed and reproducibility and long-term validity are enhanced.

## A practical example

As a proof of concept for the proposed approach, we have created a workflow where the multimodal Large Language Model GPT-4 reads a labnotebook entry, a corresponding ManGO metadata file and a publication. Using the labnotebook entry that started this project, the Metadata entry created for the image file of Fig. 1B when uploaded to the KU Leuven iRODS storage system, and this manuscript, we tested the consistency between entries using a list of five keywords. We effectively tested for the completeness of the title, the authors, the topic, the methodology and the repository used; this list is a placeholder for a checklist, a metadata standard or a published template like REMBI as mentioned above.

In the future, it would be desirable to align resources like REMBI or a recommendation list targeted at image analysis (Schmied et al, 2023) with the GPT-4 queries. We also see that this is a community effort, where metadata schemas are evolving and hopefully over time converging on a community-agreed standard. Based on our five keyword query we created a Jupyter notebook (https://github.com/vib-bic-training/Reproducibility_RDM.git), which was used to generate a report summarizing the findings of the proofreading and comparing the different entries (the exemplary labnotebook file used here as well as the metadata file can be found on the GitHub repository). We interacted with GPT4 using an API key. We also used a larger pipeline package (en_core_web_lg, 685k unique vectors) for tokenization, which could be customized towards a specific domain, such as BioImaging. The “digestion”, the text that GPT-4 found in the manuscript, the labnotebook entry, and the metadata file based on the keywords are given in Table 1. It is impressive to see that even with these five simple keywords detailed descriptions can be extracted from the sources and compared. The consistency between the entries can be visualized in the form of a heatmap per keyword and source (Fig. 1C). In a scenario where one of three data entries is different—for instance, a concentration—that value can now be corrected based on the majority of entries: the heatmap readily shows how similarity varies across the files with a “1” describing perfect similarity. Beyond the proof of concept stage and regarding the use of large language models in general, consistency over time, correctness, hallucinations, and confidence in the answer as well as the availability of the language model need to be monitored carefully for future implementations.

**Table 1:** Proof of concept report.

	Keywords				
Source	Title	Authors	Topic	Methodology	Repository
Manuscript	What we can learn from deep space communication for reproducible bioimaging and data analysis	Tatiana Woller, Christopher J. Cawthorne, Romain Raymond Agnes Slootmaekers, Ingrid Barcena Roig, Alexander Botzki, Sebastian Munck	The paper discusses the challenge of maintaining reproducibility in BioImage data analysis, much like the challenges in deep space communications. It recommends the use of AI language model proofreading to improve error correction and consequently, the fidelity of the data.	The researchers created a workflow where the Large Language Model GPT-4 reads a lab notebook entry, a corresponding ManGO metadata file, and a publication to look for a list of keywords and check them for consistency across these metadata sources. Error correction is then performed by using AI to highlight gaps and contradictions.	https://github.com/vib-bic-training/Reproducibility_RDM.git
ELN	What we can learn from deep space communication for reproducible BioImaging and data analysis	Not specified	The project aims to understand the learnings from deep space communication, which can be applied for better and reproducible BioImaging and data analysis.	The project relies on a Python-based main script which interacts with GPT-4 through an API (application programming interfaces). The user must obtain an API key from OpenAI to run the analysis. It accepts input in pdf, txt, json files format.	https://github.com/vib-bic-training/Reproducibility_RDM.git
ManGO Metadata	What we can learn from deep space communication for reproducible BioImaging and data analysis	Tatiana Woller, Christopher Cawthorne, Romain Raymond Agnes Slootmaekers, Ingrid Barcena Roig, Alex Botzki, Sebastian Munck	The study discusses what can be learned from deep space communication for making BioImaging and data analysis more reproducible.	The research utilized a large language model (GPT-4) for its methodology.	https://github.com/vib-bic-training/Reproducibility_RDM.git
All sources	“What we can learn from deep space communication for reproducible BioImaging and data analysis”	Tatiana Woller, Christopher J. Cawthorne, Romain Raymond Agnes Slootmaekers, Ingrid Barcena Roig, Alexander Botzki, Sebastian Munck.	The main focus is on improving the reproducibility of BioImage and data analysis, by drawing inspiration from deep space communication techniques. It suggests using AI language model proofreading to consolidate various metadata entries, and improve error correction, consequently enhancing the fidelity of data and its documentation, thereby paving the way for higher reproducibility and reusability.	The main methodology involves the use of a large language model AI, specifically GPT-4, to process various metadata sources such as lab notebook entries, metadata files, and publications. This AI assists in checking for consistency across these metadata sources and performs error correction by highlighting gaps and contradictions. A Jupyter notebook was created for proofreading and comparing the different entries. The AI model’s response in the form of a structured report was then used to visualize the similarities between the sources and to correct inconsistencies.	The code and workflows for the study have been made available in the following GitHub repository: “https://github.com/vib-bic-training/Reproducibility_RDM.git”.

A Jupyter notebook using GPT-4 has been used to create a structured output in the form of a Table (CSV file). The digestion of a labnotebook entry, a ManGO metadata file and this manuscript are used to check for keywords and their consistent use.

Overall, we believe that the procedure outlined here can reduce reporting errors and improve the reproducibility and FAIRness of bioimage data. Generating interpretable readouts in the form of heatmaps that highlight where metadata differs or is missing, could help to consolidate records and complete information more easily. This should improve the overall quality of reporting and future-proof the reproducibility and reusability of data for follow-up studies. Given the simplicity of the approach, it can be easily adopted, allowing image data to boldly go FAIR (where too little data has gone before).
